# Potential Bias in Assessing the Tobacco/Nicotine—COVID-19 Association—How to Improve Our Level of Understanding

**DOI:** 10.3390/ijerph192114113

**Published:** 2022-10-28

**Authors:** Ivan Berlin

**Affiliations:** Département de Pharmacologie, Hôpital Pitié-Salpêtrière-Sorbonne Université, 75013 Paris, France; ivan.berlin@aphp.fr

## 1. Introduction

The causative agents of COVID-19 are the variants of SARS-CoV-2 (severe acute respiratory syndrome coronavirus 2). The virus’s main entrance way is through mucosal tissues: the nose, mouth, upper respiratory tract, and, less frequently, conjunctival mucosa. Its gravest clinical manifestation is severe respiratory insufficiency.

Tobacco smoke exposure results in inflammatory processes in the lung, increased mucosal inflammation, the expression of inflammatory cytokines and tumor necrosis factor α, increased permeability in epithelial cells, mucus overproduction, and impaired mucociliary clearance [1]. According to the 2010 Surgeon General Report [2], the evidence is sufficient to infer a causal association between tobacco smoking, involuntary exposure to tobacco smoke, acute respiratory illnesses, and all major respiratory symptoms among adults and exposed children: cough, phlegm, dyspnea, and wheezing. Direct and indirect tobacco smoke exposure is associated with smell and odor disturbances; these are also observed with COVID-19.

Because smoking predisposes all viral, bacterial, and fungal infections, and in particular respiratory system infections [3], it is a strong hypothesis that individuals exposed to tobacco smoke by active smoking, by secondhand smoke, or having been previously exposed to smoking and consequently having smoking-related respiratory (or cardiovascular) illnesses are at increased risk of SARS-CoV-2 infections and more severe instances of COVID-19 than individuals devoid of tobacco smoke exposure.

Based on these considerations, the following questions can be raised. Compared to never smokers or never tobacco users, (a) is the risk of *catching SARS-CoV-2 infection* higher, lower, or similar with or without comorbidities, and (b) is the risk of having a more, less, or similar *severity of COVID-19 disease* higher, lower, or similar with or without comorbidities among the following:

- Current smokers, including all kinds of smoked tobacco: cigarettes, cigars, pipes, and hookahs/waterpipes;
- Current smokeless tobacco users;
- Individuals currently exposed to secondhand tobacco smoke (adults and children);
- Former smokers, including all kinds of smoked tobacco: cigarettes, cigars, pipes, hookahs/waterpipes;
- Former smokeless tobacco users;
- Individuals previously exposed to secondhand tobacco smoke (adults and children).

The same questions should be raised for users (a) of nicotine delivery systems (electronic or other nicotine delivery systems) with (dual users) or without concomitant tobacco use as well as users (b) of heated tobacco products (HTPs).

## 2. Methodological Considerations

The assessment of cause to effect is a major aim in epidemiological studies in order to help policy decisions and improve population health. The gold standard method with which to assess the cause to effect relationship in a clinical setting is a randomized controlled trial (RCT). Sufficiently powered RCTs lead to homogeneous baseline populations, making the assessment of the associations between interventions and controls acceptable because potential factors contributing to the main predefined outcomes are similarly distributed in all randomized groups, rendering the estimate of the association valid. However, for ethical reasons, this RCT-type approach cannot be applied to large, nonclinical populations, i.e., one cannot prospectively expose a large number of individuals to a potential risk comparatively to a population not exposed to this potential risk.

Usually, prospective epidemiological studies are not randomized, and even if controlling for a large number of previously established factors, unknown or not recorded factors may contribute to the outcomes, making estimations uncertain. Sufficiently powered random sampling prospective studies would allow for the control for selection bias, confounders, and collider bias (see below).

In 1965, Bradford Hill proposed a number of considerations in response to the question “What aspects of that association (that is between two variables) should we especially consider before deciding that the most likely interpretation is causation?” [4]. He listed the following characteristics of the association: strength of the association, consistency, specificity, temporality, dose–response/exposure association, plausibility, coherence, experimental or semi-experimental evidence, and analogy. He pointed out that “No formal tests of significance can answer those questions” but global reasoning applied to the findings.

The tobacco–SARS-CoV-2 infection and the tobacco–COVID-19 severity relationships are good examples of the difficulties arising from observational data.

### 2.1. Design

Most of the currently available publications on the tobacco use (essentially cigarette smoking) and SARS-CoV-2 infection or COVID-19 severity relationships are cross-sectional association studies with retrospective data collection. To the best of our knowledge, all are secondary analyses of existing observational data, either clinical or from databases that are frequently probability samples. Comparisons are usually run between current smokers, former smokers, and never smokers based on self-reports. Comparisons of data in the same samples of current, former, and never smokeless tobacco users as well as current and former nicotine users (including nicotine replacement therapy and nicotine delivery systems) with non-smokers and no tobacco or nicotine users would provide validity to the findings as to whether the association of interest is due to tobacco, smoked tobacco, or nicotine. However, only random sampling could protect against known or unknown confounders.

In several reports, the control group is constituted post hoc or consists of geographical or historical controls, making the characterization of the control group insufficient and the matching biased. Frequently, cases and controls are not matched.

### 2.2. Data Collection

Because current reports’ designs were not conceived specifically for looking at the tobacco/smoking–SARS-CoV-2 infection or COVID-19 severity relationships, the data collection lacks measures typically used in nicotine and tobacco research. Some weaknesses in the data collection/recording are listed below:

- Lack of accuracy in the assessment of smoking-related characteristics: no precise definitions of current or former smoker status—what is the time period of tobacco abstinence to consider an individual as a former smoker; no assessment of duration or intensity of smoking; and no biochemical verification of smoking status;
- Recording smoking characteristics in an emergency situation is difficult, and smokers who stopped smoking recently could be classified as former smokers;
- No assessment of secondhand smoke exposure;
- Insufficient record of previous chronic and current comorbidities. Acquiring the infection and having a more severe instance of COVID-19 is more likely among individuals with pre-existing comorbidities;
- Insufficient or no information about other substance use (e.g., alcohol use);
- Insufficient demonstration of the exposure–risk (“dose–response”) association, usually by the lack of a categorized intensity of the exposure;
- Potential over-representation of healthcare workers (they are more likely to be infected);
- Diversity of ethnicity (ethnic minorities are more likely to be infected by SARS-CoV-2);
- Testing of individuals at high risk (e.g., smokers).

### 2.3. Confounding Variables

Confounder bias occurs when the data analysis insufficiently or does not control for one or several variables that can increase the likelihood of outcomes and whose distribution by group is not random across groups. Pre-existing or acute smoking/tobacco-use-associated illnesses are confounding variables.

It is an acceptable hypothesis that smoking/tobacco-associated comorbidities contribute to or independently predict severity and mortality associated with COVID-19. This can be assessed by a mediational analysis. For example, Au Yeung et al. [5] have shown in a Mendelian randomization study that the smoking–hospitalization for and the severity of COVID-19 relationships do not involve the mediation effect of lung function or chronic obstructive pulmonary disease, lending support to the hypothesis that these two outcomes are specifically associated with smoking.

### 2.4. Collider Bias

Smokers are more likely to be tested for SARS-CoV-2 infection because the overall risk of any infection is higher among them, and because they frequently have respiratory symptoms. For example, the likelihood to perform a RT-PCR test is higher among those who are coughing. Smoking is associated with coughing. The choice to perform a test is not random, but because coughing is associated with smoking; consequently, the likelihood of a negative test among smokers is higher than among non-smokers. Inversely, coughing in a non-smoker may be a symptom of COVID-19; therefore, the likelihood of a positive test is higher among non-smokers than among smokers. This selection bias influences the outcome as a collider [6].

As opposed to random sampling, current observational data may be subject to sampling on the condition of voluntary participation (participation is strongly non-random), symptom severity and susceptibility, occupation (healthcare workers), geographical situation, and social connectedness (i.e., being aware and having access to a study) [7]. Internet access and technological capabilities are selection processes and may also introduce a collider bias [7].

The lack of the specificity of the association of tobacco use disorder with COVID-19 may indicate a common factor or factors increasing the risk of acquiring SARS-CoV-2 infection among individuals with substance use disorders [8].

### 2.5. Interpretation Bias

Not adjusting for available confounders or, inversely, controlling for a collider [9] can result in concluding about an association between exposure and outcome when, in fact, none exists.

Findings about active-smoking-related associations cannot be generalized to secondhand smoke exposure or to smokeless tobacco. Similarly, findings about tobacco-related associations cannot be generalized to nicotine.

## 3. What Would Be the Requirements to Ascertain the Cause to Effect Relationship between Exposure to Tobacco Smoke (or Tobacco) and SARS-CoV-2 Infection?

1. Prospective design with random sampling and with the primary aim of specifically looking for a tobacco/smoking–SARS-CoV-2 infection rate or COVID-19 severity association, meaning to prospectively follow up non-exposed and exposed individuals that have no signs, symptoms, or any variant of SARS-CoV-2 at baseline for a sufficiently long time period.

2. Infection positivity rates to be assessed among random samples: All individuals tested for SARS-CoV-2 regardless of the probability of infection.

3. Exhaustive data collection: To assess all biological and behavioral components associated with tobacco use, such as the intensity of exposure, previous history of tobacco exposure (age of first cigarettes, previous quit attempts, current and previous active exposure, change in smoking behavior, current and previous secondhand smoke exposure), concomitant and previous use of other substances, such as alcohol or other substances of abuse and dependence, previous and current medical and mental co-morbidities, time spent for tobacco use (exposure per day), biochemical verification of current tobacco exposure, and life style assessment (exercise, eating habits, etc.). The form of tobacco use should be assessed: cigarettes and other forms of smoked tobacco, smokeless tobacco use, and concomitant nicotine uptake. Current knowledge does not allow for the disentangling of which of the listed factors contribute to or protect against the risk of having SARS-CoV-2 infection. Besides these collectable characteristics, other characteristics, such as a genetic predisposition for tobacco’s effect on health and for immunological responses to viral infections (SARS-CoV-2), may influence the outcome.

4. To compare outcomes by two or several approaches in the same dataset: One way to overcome the fallacy of clinical data collection is the Mendelian randomization approach [10,11]. This approach is the use of genetic variants of tobacco use behaviors, one or several genetic proxies, that have previously shown strong associations with a smoking phenotype. Using genetic proxies may help in avoiding uncertainties in data collection. The main conditions of using Mendelian randomization is that the genetic marker’s validity to predict an outcome had been previously ascertained and that the genetic variants do not influence the outcome (see the list of assumptions for the validity of Mendelian randomization studies in Davies et al., 2018) [10]. However, the strength of the association between genetic proxy and tobacco use behavior is expressed as a probability estimate, and the level of error should be taken into account.

A good example is the paper by Clift et al., 2021 [12]. The authors used the UK Biobank data; COVID-19 outcomes were derived from Public Health England SARS-CoV-2 testing, hospital admissions, and death certificates. The multivariable regression fully adjusted model showed that the risk of SARS-CoV-2 infection did not differ between never smokers and current smokers, but that former smokers had a higher risk of infection than never smokers and that hospitalization as well as mortality were higher in both former and current smokers. Contrarily to the results of the multivariable regression analysis, the results from the Mendelian randomization data showed that individuals with genetic variants of smoking initiation or smoking heaviness had a higher risk of a confirmed infection. Results for the risk of hospitalization and death rate were similar by using the two different approaches. Clift et al.’s [12] Mendelian randomization data were later confirmed by Au Yeung et al. [5] using the lifetime smoking index [13].

5. RCTs could answer the question as to whether nicotine itself is associated with the likelihood of SARS-CoV-2 infection, progression, and severity, but recruitment may be a major challenge. RCTs of vaccines against COVID-19 could provide information, as a secondary aim, about the contribution of smoking/tobacco/nicotine use if these participants’ characteristics were recorded and followed up. This could be the easiest, quickest, most cost-effective, and most reliable way to estimate the contribution of tobacco/nicotine use in preventing or not preventing COVID-19.

6. Most importantly, the preregistering of data analysis plans and causality hypotheses using acyclic graphs may provide more confidence in the results [11]. Preregistration increases the distinction between hypothesis generation and hypothesis testing, as well as increasing the credibility of research findings [14].

## 4. Regulatory Considerations

According to currently available data, the role of smoking, tobacco, or other nicotine inhalation delivery systems’ use in acquiring SARS-CoV-2 infections is not established. In contrast, the harmful effect of tobacco smoking on the severity and mortality of COVID-19 is well-established [5,12,15,16,17,18,19]. This means that current regulatory strategies [20] to fight the tobacco epidemic should not be changed.

## 5. Policy Gaps and Recommendations for Further Work

Stopping smoking, and also probably all tobacco and inhaled nicotine use, may reduce SARS-CoV-2-associated disease severity. This can be an argument to quit any tobacco or electronic cigarette use during the COVID-19 pandemic.

We would need prospective data about NRT, waterpipe, and HTP use in association with acquiring SARS-CoV-2 infections and associated disease severity as well as mortality. Because of the increasing worldwide use of various nicotine delivery systems, data on their use and the SARS-CoV-2 infection rate in addition to COVID-19 severity are urgently needed; they may vehicle infectious agents, deliver products in the upper and lower respiratory tract, and may cause respiratory symptoms with but also without concomitant smoking.

## References

[1] Strzelak A., Ratajczak A., Adamiec A., Feleszko W. (2018). Tobacco smoke induces and alters immune responses in the lung triggering inflammation, allergy, asthma and other lung diseases: A mechanistic review. Int. J. Environ. Res. Public Health.

[2] Centers for Disease Control and Prevention (US), National Center for Chronic Disease Prevention and Health Promotion (US), Office on Smoking and Health (US) (2010). Atlanta (GA): Centers for Disease Control and Prevention (US). https://www.ncbi.nlm.nih.gov/books/NBK53021/#_NBK53021_pubdet_.

[3] Arcavi L., Benowitz N.L. (2004). Cigarette smoking and infection. Arch. Intern. Med..

[4] Hill A.B. (1965). The environment and disease: Association or causation?. Proc. R. Soc. Med..

[5] Au Yeung S.L., Li A.M., He B., Kwok K.O., Schooling C.M. (2022). Association of smoking, lung function and COPD in COVID-19 risk: A two-step Mendelian randomization study. Addiction.

[6] Tattan-Birch H., Marsden J., West R., Gage S.H. (2021). Assessing and addressing collider bias in addiction research: The curious case of smoking and COVID-19. Addiction.

[7] Griffith G.J., Morris T.T., Tudball M.J., Herbert A., Mancano G., Pike L., Sharp G.C., Sterne J., Palmer T.M., Davey Smith G. (2020). Collider bias undermines our understanding of COVID-19 disease risk and severity. Nat. Commun..

[8] Wang Q.Q., Kaelber D.C., Xu R., Volkow N.D. (2021). COVID-19 risk and outcomes in patients with substance use disorders: Analyses from electronic health records in the United States. Mol. Psychiatry.

[9] Lee H., Aronson J.K., Nunan D. (2019). Catalogue of Bias Collaboration. Collider Bias. Catalogue of Bias.

[10] Davies N.M., Holmes M.V., Davey Smith G. (2018). Reading Mendelian randomisation studies: A guide, glossary, and checklist for clinicians. BMJ.

[11] Perski O., Simons D., Shahab L., Brown J. (2022). Smoking, nicotine and COVID-19: Triangulation of methods and pre-registration are required for robust causal inference. Nicotine Tob. Res..

[12] Clift A.K., von Ende A., Tan P.S., Sallis H.M., Lindson N., Coupland C.A.C., Munafò M.R., Aveyard P., Hippisley-Cox J., Hopewell J.C. (2022). Smoking and COVID-19 outcomes: An observational and Mendelian randomisation study using the UK Biobank cohort. Thorax.

[13] Leffondré K., Abrahamowicz M., Xiao Y., Siemiatycki J. (2006). Modelling smoking history using a comprehensive smoking index: Application to lung cancer. Stat. Med..

[14] Nosek B.A., Ebersole C.R., DeHaven A.C., Mellor D.T. (2018). The preregistration revolution. Proc. Natl. Acad. Sci. USA.

[15] Zhao Q., Meng M., Kumar R., Wu Y., Huang J., Lian N., Deng Y., Lin S. (2020). The impact of COPD and smoking history on the severity of COVID-19: A systemic review and meta-analysis. J. Med. Virol..

[16] Reddy R.K., Charles W.N., Sklavounos A., Dutt A., Seed P.T., Khajuria A. (2021). The effect of smoking on COVID-19 severity: A systematic review and meta-analysis. J. Med. Virol..

[17] Hou H., Li Y., Zhang P., Wu J., Shi L., Xu J., Diao J., Wang Y., Yang H. (2021). Smoking Is Independently Associated With an Increased Risk for COVID-19 Mortality: A Systematic Review and Meta-analysis Based on Adjusted Effect Estimates. Nicotine Tob. Res..

[18] Simons D., Shahab L., Brown J., Perski O. (2021). The association of smoking status with SARS-CoV-2 infection, hospitalization and mortality from COVID-19: A living rapid evidence review with Bayesian meta-analyses (version 7). Addiction.

[19] Patanavanich R., Siripoon T., Amponnavarat S., Glantz S.A. (2022). Active smokers are at higher risk of COVID-19 death: A systematic review and meta-analysis. Nicotine Tob. Res..

[20] WHO Q & A on Tobacco, Waterpipe and E-Cigarette Use in the Context of COVID-19. World Health Organization. Regional Office for the Eastern Mediterranean. https://apps.who.int/iris/handle/10665/342027.

